# Prevalence of Overweight and Obesity in Children and Adolescents from the Age Range of 2 to 19 Years Old in Brazil

**DOI:** 10.1155/2014/583207

**Published:** 2014-06-03

**Authors:** Janaina R. Niehues, Ana Inês Gonzales, Robson R. Lemos, Poliana Penasso Bezerra, Patrícia Haas

**Affiliations:** ^1^Division of Health Sciences, Núcleo de Pesquisa e Desenvolvimento da Saúde (NUPEDS), Universidade Federal de Santa Catarina, 88900-000 Araranguá, SC, Brazil; ^2^Division of Sport and Medicine, Núcleo de Cardiologia e Medicina do Exercício (CEFID), Universidade Estadual de Santa Catarina, 88080-350 Florianópolis, SC, Brazil

## Abstract

*Introduction*. Infant juvenile obesity is currently a worldwide public health problem and it is increasing at alarming rate in the Brazilian population, showing its relevance in terms of public health. *Objectives*. Determine the prevalence of overweight and obesity in children and adolescents between 2 and 19 years old in different regions of Brazil. *Methods*. The following electronic databases were searched (from September through November 2013): Medline (PubMed), LILACS, and SciELO, using the descriptors and Boolean operators (*obesity*) and (*overweight*) and (*child*) and (*prevalence*) and (*Brazil*). Prospective and/or cross-sectional designs studies were found regarding the prevalence of overweight and obese children and adolescents in the five regions of Brazil. *Results*. A total of 191 scientific articles were found of which 17 met all inclusion criteria. A higher prevalence of overweight was found in the south (25.7%) and north (28.8%) of the country, and obesity in the southeast (15.4%) and south (10.4%). *Conclusions*. The scope of the studies was mostly based on municipal coverage, which resulted in limitations for conclusive analysis, showing the need for further studies of prevalence at the national level, with emphasis on public health in obese children and adolescents throughout the Brazilian territory.

## 1. Introduction

According to the World Health Organization (WHO), obesity can be defined as the accumulation of body fat in an abnormal and/or excessive manner showing serious health problems. In this scenario, overweight and obesity are considered a serious public health problem, and it is therefore a subject of considerable impact and worldwide interest [1].

This particular condition characterized by increased adipose tissue from the positive energy calculation in the relation intake versus calorie expenditure has a multifactorial etiology; among these we can highlight genetic susceptibility, metabolic disorders, sex, age, occupation, diet, and others [2, 3].

The concern with this health condition is becoming increasingly clear since the exponential increase in its prevalence and incidence, due to declining levels of physical activity and increased inadequate food intake [4–6]. It is estimated that the worldwide prevalence of obesity in the period between 1980 and 2008 has doubled; today it is estimated that worldwide approximately 2.8 million annual deaths are related to the harmful effects of excess weight [1, 7–9], raising global public spending as a result of this condition [10, 11].

Until recently, obesity was considered a problem only in developed countries; however, it is currently on the rise in developing countries [1, 7–9]. In Brazil, the overweight and obesity are on the rise among children and adolescents, mainly after the age of five, in all economic classes and in all regions. Between 2008 and 2009 the excess weight reached 33.5% of children from five to nine years old and within this group 16.6% of boys were obese, while 11.8% of girls were obese. Overweight was more common in urban areas compared to rural areas [12].

Overweight in childhood predisposes the short- and long-term comorbidities such as diabetes mellitus, hypertension, and dyslipidemia [13]. Since children are still in their infancy, early control is necessary at this stage of life, in order to avoid an unfavorable long-term prognosis, as in adulthood. Future complications of this condition in adulthood can be serious if early intervention measures are not established [14].

In this context, it is essential to identify the prevalence of obesity and overweight children and adolescents in Brazil and in which regions of the country [14]. For this reason, the aim of this systematic review is to determine the prevalence of overweight and obesity in children and adolescents from the age range of 2 to 19 years old in different regions of Brazil.

## 2. Materials and Method

A systematic review was conducted according to the recommendations of the* Preferred Reporting Items for Systematic Reviews and Meta-Analyses (PRISMA)* [15].

### 2.1. Inclusion and Exclusion Criteria

This review included prospective and/or cross-sectional designs studies on the prevalence of overweight and obese infant juvenile in different regions of Brazil. Inclusion criteria were as follows: Brazilian studies, with subjects aged 2–19 years [16], considered obese and/or overweight [17].

There was no language restriction for the search, and all included studies were translated where necessary and possible. Inadequate or poorly described interventions were considered as exclusion criteria.  Table 1 provides a summary of inclusion and exclusion criteria of this review.

### 2.2. Search Strategies

The search for relevant scientific articles was conducted by independent researchers in electronic databases such as Medline (PubMed), LILACS, and SciELO from September through November 2013. The search was structured as PICO, acromion for target patient, intervention, control, and outcome. The search was based on the words of the dictionary* Medical Subject Heading Terms* (MeSH), descriptors, and Boolean operators. The first search was conducted in PubMed database as follows: ((*obesity*), and (*overweight*) and (*child*) and (*prevalence*) and (*Brazil*)). The searches in the subsequent databases were adjusted according to the specifications needed for the databases keeping similar words in the search process. In order to complement the search process, a manual search was performed of the references included in the articles found in the databases.

### 2.3. Selection of Studies

Two independent observers analyzed the results to find potentially eligible studies. Initially, the studies were selected according to the title, then the abstracts were reviewed, and only those which were potentially eligible were selected. Based on the abstracts, full articles were acquired for the final analysis. In case of disagreement between reviewers, a third reviewer made the decision on the eligibility of the study.

## 3. Results and Discussion

### 3.1. Results

**Figure 1:**
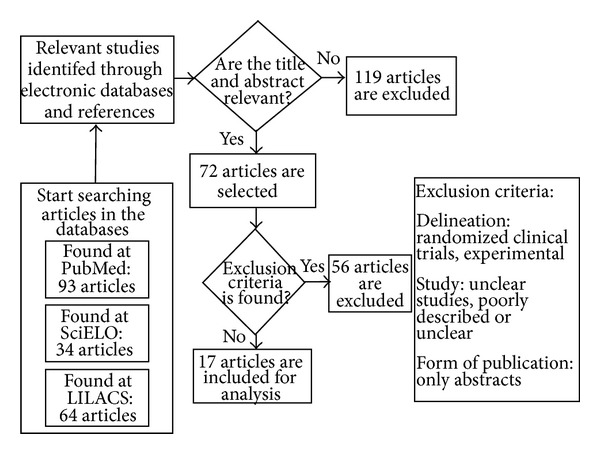
Flowchart of the search process.

A total of 191 articles were identified in the search (Figure 1); 72 were selected for evaluation in accordance with the title and their revised abstracts. Based on these eligible articles for a full review, a total of 17 articles met all the proposed inclusion criteria.

From the 17 articles identified in this systematic review, all used cross-sectional designs. When verified the scope of the studies, 16 [18–34] were carried out at the municipal level, just [33], and one particular study was carried out statewide. After searching the databases, we notice that no studies have been undertaken at the national level.

**Figure 2:**
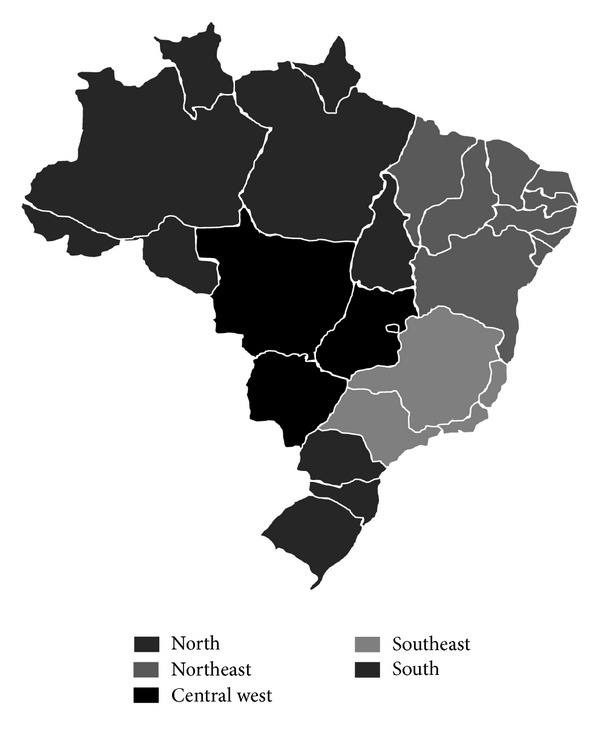
Political map of Brazil.

When the geographic regions are observed, studies for seven articles were performed in the south of Brazil (states of Rio Grande do Sul, Santa Catarina, and Paraná), five in the southeast (states of São Paulo, Rio de Janeiro, Espirito Santo, and Minas Gerais), three in the northeast region (states of Maranhão, Piauí, Rio Grande do Norte, Ceará, Paraíba, Pernambuco, Alagoas, Bahia, and Sergipe), one in the north region (states of Acre, Amazonas, Rondônia, Roraima, Amapá, Pará, and Tocantins), and one in the central west of Brazil (states of Mato Grosso, Mato Grosso do Sul, Goiás, and Distrito Federal) (Figure 2) (Table 2).

Taking into account the year of publication, there was an increase in the number of articles published in the last five years. Likewise, it is important to point out that there was an exponential increase in the number of individuals evaluated (5,889 in the period from 2003 to 2008 and 10,227 in the period from 2009 to 2012).

In different regions of Brazil, there was a variation in the prevalence rates of overweight and obesity. In the south, the rates were approximately 25.7% and 10.4%, respectively, with subjects aged 6–18 years. In the southeast, rate of overweight was 13.7% and obesity 15.4%, with subjects aged 2–19 years. In the northeast region, rate of overweight was 15.8% and obesity 4.3% with population aged 6–19 years. In the north, the only study found showed a prevalence of 28.8% overweight with population aged 6–19 years. Likewise, the only study in the central west region showed a prevalence of overweight of 16.8% and 5.3% obesity in children aged 6–10 years.

### 3.2. Discussion

The results of this study demonstrated that a higher prevalence of overweight was found in the south (25.7%) and northeast (28.8%) of the country, as well as a higher prevalence of obesity in the southeast (15.4%) and south (10.4%). Note that only one study was conducted in the north region, showing the limitation of this particular finding. However, regardless of the region in which different studies had been conducted, a high prevalence of overweight and obesity in Brazilian children and adolescents was identified.

Currently, the increasing prevalence of obesity and overweight in children and adolescents is observed worldwide and it has effects on the status of health and quality of life. In Brazil, data from the National Demographic and Health Survey indicated that 7.3% of children under 5 years old are overweight [35]. Another national study found that one in three children aged 5 and 9 years are overweight, according to the guidelines of the World Health Organization [12]. A survey conducted in the period from 2008 to 2009 by the Brazilian Institute of Geography and Statistics in partnership with Ministry of Health showed that the prevalence of overweight among children aged 5–9 years increased from approximately 13.4% in 1989 to 33.4% in 2008. Likewise, obesity increased from 3.2% to 14.2% [12].

Regarding adolescents, the same survey in 1989 showed a prevalence of overweight of 10.8% and obesity of 1.3%, in which the rates were increased to 20.5% and 4.9%, respectively.

In a study realized in the United States, in the period from 1999 to 2012, it was revealed that 17.3% of children were obese, and 5.9% and 2.1% were in obesity classes 2 and 3, respectively [36]. Another survey showed that, in the age group 2–19 years old, 16.9% of North Americans were obese [37]. In Latin America, a systematic review showed that between 18.9% and 36.9% of children of school age (5–9 years old) and between 16.6% and 35.8% of adolescents (12–19 years old) are obese. In this particular study it is estimated that 20% to 25% of children and adolescents between 5 and 19 years old are affected by obesity [38].

These findings demonstrate the clinical and epidemiological relevance of obesity in the context of public health in Brazil and in the world as it has become a global epidemic, directly affecting the world population [39].

The development of juvenile obesity is related to eating habits, level of physical activity, sedentary practices, socioeconomic status, and genetics, among others [40–42]; however, it is attributed to the increase of overweight in children and adolescents, observed in recent decades, the concomitant decline in levels of physical activity, and increased eating inappropriate behaviors [43–45] significant effects on body composition of these individuals. Studies included in this review demonstrated an association between overweight and inactivity in children and young Brazilians, ranging from 39% to 84.4% [18, 29]. These findings can be explained by the fact that a sedentary lifestyle is currently facilitated by technological advances (e.g., computers, television, and video games), which make no need for children to struggle physically, unlike some years ago. For fear of urban violence and by the request of the parents, staying indoors with activities that do not encourage them to do physical activities such as running, playing ball, and playing hide and seek results in spending most of the time in “sedentary” [18, 29].

The fast foods are adopting marketing strategies, aiming to capture the preference of the infantile public, and became immensely popular in Brazil [46]. It is believed that the time spent watching television and using the computer and video games is an indicator of sedentary behavior that is associated with obesity [47]. Also, the time spent on these activities is aggravated by excessive calorie intake and by minimum nutritious food intake, often induced by the media, because the children are mainly exposed to unhealthy food advertising on television [48]. Our findings corroborate such information once they have demonstrated a strong association between poor eating habits of children and young Brazilians and overweight.

It is important to highlight that the presence of a sedentary life in obese Brazilian children and adolescents is an important risk factor, since physical inactivity has been directly related as a decisive factor in the current global epidemic of overweight and obesity in all age groups [45, 49].

In developing countries, economic factors strongly influence the determination of the prevalence of overweight and obesity in a superior way compared to biological determinants [50, 51]. Young Brazilians have a higher prevalence of overweight when residing in urban areas, higher family income, and higher socioeconomic status, which is in line with our findings [52].

It is well known that the pathological process of obesity may result in consequences such as the short-term ones in cardiovascular and metabolic system as hypertension, hypercholesterolemia, cardiovascular dysfunction, insulin resistance, diabetes mellitus type 1, and atherosclerosis. In the same manner, the long-term consequences include the persistence of obesity into adulthood with associated comorbidities, including cardiovascular disease, diabetes type 2, and premature death [13, 53].

From the 17 articles selected, seven of them [19, 21, 22, 25, 26, 30, 33] also presented the information on the prevalence of hypertension and dyslipidemia associated with overweight and obesity in Brazilian children and adolescents at the municipal level. In relation to hypertension, studies have been conducted in the south region (*n* = 2), southeast (*n* = 2), and northeast (*n* = 1) with prevalence that may reach values of up to 13.6% in the northeast [26], 13.5% in the south [16], and 11.7% in the southeast [20]. Only two studies investigated the values of dyslipidemias, one in the southeast [26] and another one in the north [33], and both showed increased levels of triglycerides, total cholesterol, and low density lipoprotein (LDL) and reduced levels of high density lipoprotein (HDL).

Although a small number of studies [19, 21, 25, 26, 30, 33] have addressed the cardiovascular and metabolic comorbidities in this population, the results showed significant prevalence of hypertension and hypercholesterolemia in young Brazilians. For being children and adolescents, borderline and/or high values for systolic blood pressure (SBP) and diastolic blood pressure (DBP) predispose greater risk of developing cardiovascular problems later in adulthood [21].

In this context, changes in lifestyle, involving a combination of diet and physical activity are essential elements in the management of juvenile obesity [17] and recommended by national and international entities [20].

Moreover, emergency strategies for Brazilian children and adolescents, as well as the development of overweight and obesity prevention programs, avoiding the associated diseases, are essential, reducing the economic impacts of this condition [54, 55] since in addition to being seen as a major public health problem, obesity is responsible for the high financial cost in the global economy [10, 11]. Based on that, there is a growing worldwide concern because over the years the country will face the economic impact of the increasingly high incidence of this condition, mainly due to the comorbidities associated with the disease [56–58].

In this context, the supply of information on the major health problems of the population to the decision makers can support the development of the field of public health policy directed to this condition. Furthermore, such information should be aligned with the growing concern for the best achievable outcome in terms of public health policies [59].

The limitations of the findings in this study may have been attributed to the existence in the systematic review of only cross-sectional designs studies, which are characterized by not involving periodical monitoring of individuals, and could be useful in better establishment of associations between factors risk. In addition, cross-sectional designs studies do not allow measuring any changes in eating behavior and lifestyle [18–34].

In developing countries, studies on the juvenile obesity are still limited. Our systematic review showed a reduced number of prevalence studies in different Brazilian regions with the absence of nationwide studies, limiting a more conclusive statement on the prevalence of this public health problem that affects Brazil.

Finally, regardless of the country and its regional divisions, it is a must for all parents, educators, and health professionals to ensure the health of children and adolescents with overweight by attitudes that are consistent with established guidelines in order to promote health and reduce morbidity and mortality, trying thus to reverse the alarming prevalence rates expected to rise up.

## 4. Conclusions

In this systematic review, only 17 articles contemplated all inclusion criteria established in this study and demonstrated a higher prevalence of overweight and obesity in the south, southeast, and northeast regions of Brazil. The investigated studies were mostly municipal scope, which limited conclusive analysis on this subject.

The gap in the literature became evident, showing the need for further studies of prevalence at the national level, with emphasis on public health in obese children and adolescents throughout the Brazilian territory. Thus, it will be possible to obtain more direct and specific actions for the regions of Brazil in need of assistance as a result of this global epidemic that is spreading alarmingly in the Brazilian territory.

## Figures and Tables

**Table 1:** Inclusion and exclusion criteria.

Inclusion criteria	
Delineation	Prospective studies and/or cross-sectional designs of prevalence
Patients	Obesity and/or overweight
	Aged 2–19 years
Location	Brazilian regions (south, southeast, central west, north, and northeast)
Language	No restriction

Exclusion criteria	

Delineation	Randomized clinical trials, experimental
Study	Unclear studies, poorly described or unclear
Form of publication	Only abstracts

**Table 2:** Description of the studies included in this review in the regions of Brazil, regarding the prevalence of overweight and obesity and associated comorbidities.

Author	Year	Sample	Age	State	Overweight	Obesity
South region						
Silva et al. [18]	2012	601	14–17	Santa Catarina	16.7%	Not available
Reuter et al. [19]	2012	414	7–17	Rio Grande do Sul	Male: 22.3%	Male: 4.7%
					Female: 22.6%	Female: 12.6%
Mello et al. [20]	2010	356	6–10	Paraná	20.2%	7.0%
Burgos et al. [21]	2010	1666	7–17	Rio Grande do Sul	19.0%	7.7%
Cimadon et al. [22]	2010	590	9–18	Rio Grande do Sul	24.6%	NA
Triches and Giugliani [23]	2005	573	8–10	Rio Grande do Sul	Male: 17.3%	Male: 7.4%
					Female: 16.6%	Female: 7.6%
Terres et al. [24]	2006	1.000	15–18	Rio Grande do Sul	20.9%	5.0%
Southeast region						
Mazaro et al. [25]	2011	680	7–11	São Paulo	13.1%	9.0%
Pereira et al. [26]	2009	494	2–19	São Paulo	Male: 8.6%	Male: 19.2%
					Female: 8.4%	Female: 10.5%
Pinto and Oliveira [27]	2009	29	2–5	São Paulo	5.1%	8.2%
Fagundes et al. [28]	2008	218	6–14	São Paulo	16.5%	14.7%
Mondini et al. [29]	2007	1.010		São Paulo	17.0%	Not available
Northeast region						
Queiroz et al. [30]	2010	750	6–9	Paraíba	17.7%	3.8%
Tassitano et al. [31]	2009	4.210	14–19	Pernambuco	11.5%	2.4%
Nunes et al. [32]	2007	588	10–19	Paraíba	18.3%	6.8%
North region						
Ribas and Silva [33]	2009	437	6–19	Pará	28.8%	Not available
Central west region						
Giugliano and Carneiro [34]	2004	2.500	6–10	Distrito Federal	16.8%	5.3%

NA: not analyzed; BP: blood pressure; ↑ increase; ↓ decrease; LDL: low density lipoprotein; HDL: high density lipoprotein.
